# Dual contributions of noradrenaline to behavioural flexibility and motivation

**DOI:** 10.1007/s00213-018-4963-z

**Published:** 2018-07-11

**Authors:** Caroline I. Jahn, Sophie Gilardeau, Chiara Varazzani, Bastien Blain, Jerome Sallet, Mark E. Walton, Sebastien Bouret

**Affiliations:** 1 Motivation, Brain and Behavior Team, Institut du Cerveau et de la Moelle Epinière, CNRS UMR 7225 - INSERM U1127 - UPMC UMR S 1127, Hôpital Pitié-Salpétrière, 47, Boulevard de l’Hôpital, 75013 Paris, France; 2 Sorbonne Paris Cité Universités, Université Paris Descartes, Frontières du Vivant, 75005 Paris, France; 3 Department of Experimental Psychology, Oxford, OX1 3UD UK; 4 Wellcome Centre for Integrative Neuroimaging, University of Oxford, Oxford, UK; 5 Institute for Translational Neuroscience of Paris IHU-A-ICM, 75013 Paris, France; 6 Centre d’Economie de la Sorbonne, Université Paris 1, 75013 Paris, France

**Keywords:** Noradrenaline, Behavioral flexibility, Motivation, Clonidine, Monkey

## Abstract

**Introduction:**

While several theories have highlighted the importance of the noradrenergic system for behavioral flexibility, a number of recent studies have also shown a role for noradrenaline in motivation, particularly in effort processing. Here, we designed a novel sequential cost/benefit decision task to test the causal influence of noradrenaline on these two functions in rhesus monkeys.

**Methods:**

We manipulated noradrenaline using clonidine, an alpha-2 noradrenergic receptor agonist, which reduces central noradrenaline levels and examined how this manipulation influenced performance on the task.

**Results:**

Clonidine had two specific and distinct effects: first, it decreased choice variability, without affecting the cost/benefit trade-off; and second, it reduced force production, without modulating the willingness to work.

**Conclusions:**

Together, these results support an overarching role for noradrenaline in facing challenging situations in two complementary ways: by modulating behavioral volatility, which would facilitate adaptation depending on the lability of the environment, and by modulating the mobilization of resources to face immediate challenges.

**Electronic supplementary material:**

The online version of this article (10.1007/s00213-018-4963-z) contains supplementary material, which is available to authorized users.

## Introduction

Noradrenaline is among the most widespread neuromodulators in the brain. It was initially associated with vigilance and arousal (Kety 1972; Harley 1987; Aston-Jones et al. 1991; Berridge 1991; Waterhouse et al. 1998; Berridge and Waterhouse 2003), but several authors suggested that this role could extend to cognitive functions such as attention, learning, and memory (Usher et al. 1999; Arnsten 2000; Harley 2007; Arnsten et al. 2012; Sara and Bouret 2012; Sara 2015) and with a particular emphasis on various forms of behavioral flexibility (Devauges and Sara 1990; Bouret and Sara 2004; Chamberlain et al. 2006; Tait et al. 2007; McGaughy et al. 2008; Guedj et al. 2016). This led several authors to put behavioral flexibility at the heart of the functional role of noradrenaline in cognition (Yu and Dayan 2005; Aston-Jones and Cohen 2005; Bouret and Sara 2005). More recently, we and others have also emphasized the potential role of noradrenaline in motivation, with a strong role in effort processing (Ventura et al. 2008; Bouret and Richmond 2009; Zénon et al. 2014; Varazzani et al. 2015).

While aspects of these theories overlap, it has nonetheless been difficult to determine how to reconcile these different ideas as they have seldom been directly tested in the same experiment. A further complication is the strong relation between autonomic arousal and cognitive functions, classically attributed to the noradrenergic system (Einhäuser et al. 2008; Preuschoff et al. 2011; Nassar et al. 2012; Gee et al. 2017). This is based on the correlation between locus ceruleus (LC) firing and pupil diameter, even if this relation is far from being specific. Indeed, LC activity correlates with multiple measures of arousal, and these measures of autonomic arousal are associated with the activation of several other brain regions (Abercrombie and Jacobs 1987; Berridge and Waterhouse 2003; Joshi et al. 2016; Gee et al. 2017). Thus, one hypothesis would be that noradrenaline is simply associated with autonomic arousal and contributes to all processes linked with arousal in a highly non-specific fashion. In that frame, the various functions classically attributed to noradrenaline (behavioral flexibility and motivation), and measures associated with these (e.g., response latencies, willingness-to-work, effort sensitivity, force production or patterns of choices), would all co-vary across states of arousal and/or vigilance. Alternatively, noradrenaline could have separable influences on specific and independent processes, over and above its influence on vigilance and arousal.

The goal of this experiment was to test the causal role of noradrenaline in behavioral flexibility and motivation using a quantitative approach. Behavioral flexibility, the adaptation of the behavior to changes in the environment, could be achieved through two distinct (but non-exclusive) classes of processes: a high level executive control of the behavior (directed exploration) and a low level change of variability in the behavior (random exploration), which would allow the animal to randomly sample several alternatives. To do this, we developed an original sequential cost-benefit decision-making task, used computational modeling to identify precisely the cognitive processes of interest, and then examined the consequences of manipulating central noradrenergic neurotransmission using systemic injection of clonidine, an alpha-2 noradrenergic receptor agonist, which has been shown to decrease the firing of LC neurons and reduce central noradrenaline levels (Abercrombie and Jacobs 1987; Abercrombie et al. 1988; Grant et al. 1988; Berridge and Abercrombie 1999; Bouret and Richmond 2009).

Clonidine had two distinct effects. First, in line with the role of noradrenaline in the simplest aspect of behavioral flexibility, clonidine dose-dependently decreased choice variability: under clonidine, monkeys’ choices became more consistent with the cost-benefit analysis, but did not change their overall switch probabilities. Second, in line with the putative role of noradrenaline in motivation and effort, clonidine dose-dependently reduced force production during the task, though left animals’ willingness-to-work and cost-benefit trade-offs unaffected. Importantly, these effects on choice variability and force production did not co-vary across drug doses, as one would expect if they were simply driven by fluctuations of arousal/vigilance.

## Materials and methods

### Monkeys

Two male rhesus monkeys (monkey A, 15 kg, 5 years old; monkey D, 15 kg, 6 years old) and one female (monkey E, 4.5 kg, 3 years old) were used for the experiment. Their access to water was regulated. During testing days (Monday to Friday), they received water as reward and they received water according to their physiological needs over the weekend. All experimental procedures were designed in association with the Institut du Cerveau et de la Moelle Epiniere (ICM) veterinarians, approved by the Regional Ethical Committee for Animal Experiment (CREEA IDF no. 3), and performed in compliance with the European Community Council Directives (86/609/EEC).

### Task

Each monkey sat in a primate chair positioned in front of a monitor on which visual stimuli were displayed. Two electronic grips (M2E Unimecanique, Paris, France) were mounted on the chair at the level of the monkey’s hands. Monkeys were not constrained to use one hand or the other to squeeze the grips. Each grip corresponded to one side of the screen. Water rewards were delivered from a tube positioned between the monkey’s lips. Behavioral paradigm was controlled using the REX system (NIH, MD, USA) and Presentation software (Neurobehavioral systems, Inc., CA, USA).

**Fig. 1:**
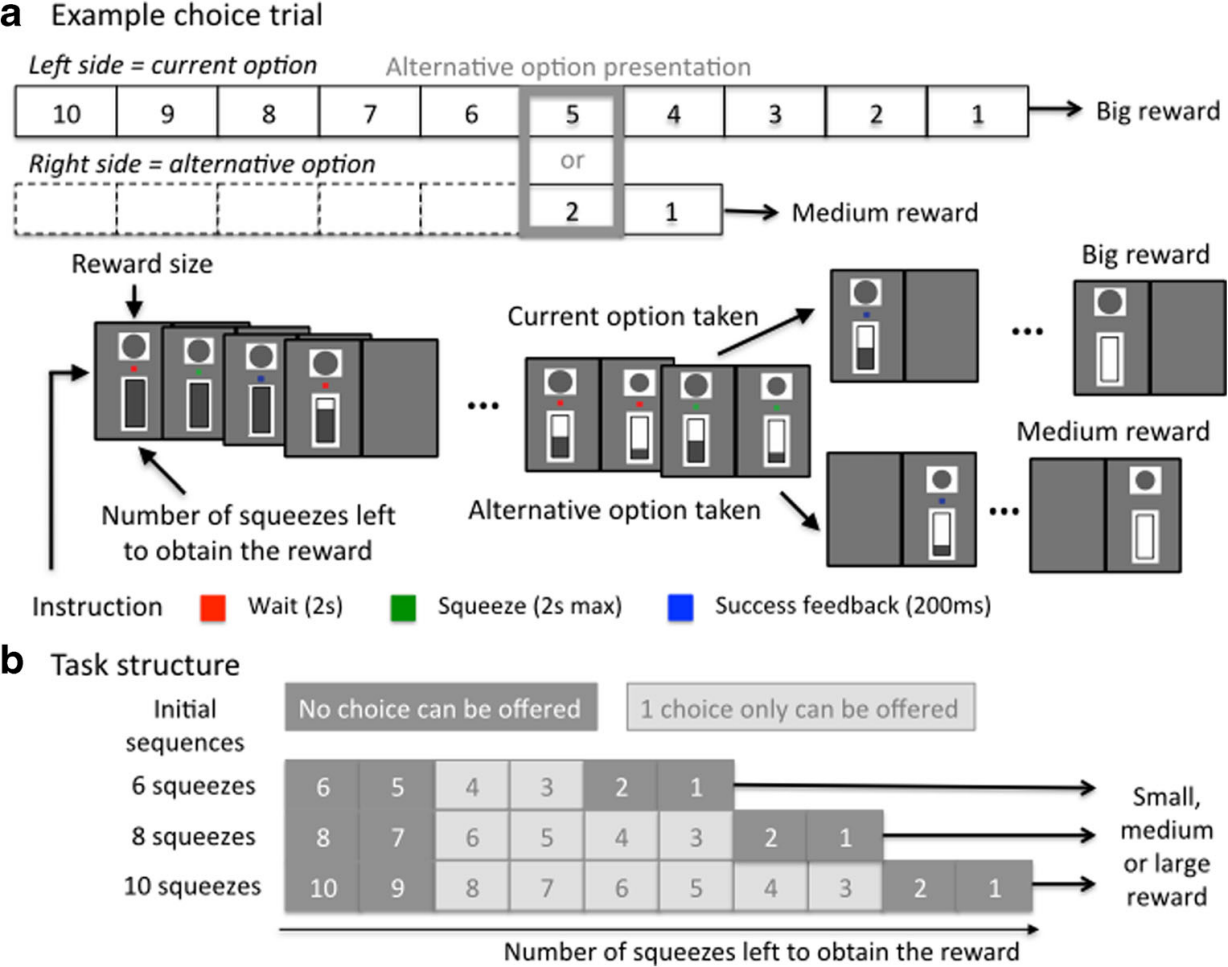
Task. Monkeys performed an operant task where they have to exert a certain number (sequence) of squeezes on a grip to obtain fluid reward. They were sitting in a chair with two grips (right and left) facing a screen. The principles of the task are: use the grip corresponding to the side where stimuli are displayed, wait when the dot is red, squeeze when it is green, and a blue dot indicates a correct squeeze. All squeezes of a sequence must be performed correctly to obtain the reward. A squeeze is incorrect if monkeys do not squeeze above the minimum force threshold when the green dot is displayed, squeeze when the red dot is displayed, or use the wrong grip. After an incorrect squeeze, the same trial restarts. After all squeezes of a sequence are performed correctly, monkeys receive the fluid reward and a new trial starts. In 70% of trials, monkeys have the choice to continue with their current sequence by using the same grip or changing grip and perform an alternative sequence for an alternative reward size. **a** Example of a choice trial (70% of trials). The trial starts with the presentation of the option, which is defined by a side (here left), an initial sequence length (10 squeezes here, bottom cue), and a reward size (big here, top cue). At each squeeze, the bottom cue indicates the remaining number of squeezes to perform (bottom cue). Five squeezes before the end of the current, an alternative option is offered. Here, by squeezing the left grip, the monkey  chooses the current option and must perform the five remaining squeezes to obtain the big reward (top). By squeezing the right grip, the  monkey chooses the alternative option and must perform two squeezes to obtain the medium reward (bottom). After all squeezes of a sequence are performed correctly, the gauge indicating the remaining number of squeezes in the sequence appears as empty and monkeys receive the fluid reward. After an inter-trial interval of 1 to 1.5 s, another trial starts. **b** Task structure. Initial sequences start with six, eight, to ten squeezes and lead to three sizes of reward (small, medium, and big). In 30% of trials, no choice is offered and monkeys must perform the initial sequence to be rewarded. In 70% of trials, one choice is offered during one of the squeeze in light gray (at least three trials after the beginning of a sequence and three trials before the end) on the figure

The task consisted of performing sequences of squeezes on a grip to obtain rewards, delivered at the end of each sequence of squeezes (Fig. 1). At the beginning of each trial, the length of the sequence (number of squeezes) and the size of the reward were indicated by two different cues that appeared simultaneously with a red dot on either the left or right side of the screen (counterbalanced across trials) (Fig. 1). There were nine initial options defined by three initial sequence lengths (six, eight, and ten squeezes) and three reward sizes (small, medium, and big). After a fixed delay of 2 s, the red dot turned green, and to initiate a trial, monkeys had 2 s to perform a squeeze above the minimum force threshold with the grip corresponding to the side of the screen where stimuli were displayed. The threshold was manually calibrated during the training phase to be minimal, meaning that monkeys would always reach the threshold if they tried to squeeze the grip (bell-shaped force profile). After a correct squeeze, the dot turned blue for 200 ms. Then, the dot turned red again and the cue corresponding the sequence length changed to show the number of remaining squeezes to complete the sequence. After an incorrect squeeze, the stimuli disappear and the same trial restarted from the beginning of the sequence after 1–1.5 s of inter-trial interval delay. A squeeze was incorrect if monkeys squeezed the wrong grip, squeezed any grip when the dot was red or did not reach the minimum force threshold 2 s after the dot turned green. In other words, trials where monkeys made an error or did not engage were repeated. After the last correct squeeze of the sequence, the dot disappeared, the cue indicating the number of remaining squeezes was at zero, and monkeys received the size of the reward corresponding to the reward cue. At the end of the reward delivery, a new trial started after 1–1.5 s of inter-trial interval delay.

In 30% of trials, monkeys were presented with no alternatives and so had to complete the initial sequence to gain the reward. However, in 70% of trials, monkeys were given the choice during the sequence to take an alternative option (Fig. 1a). The alternative option was presented on the opposite side of the screen and occurred at least three squeezes after the beginning of the initial sequence and at most three squeezes before the end of it (Fig. 1b). To choose this option, monkeys had to switch to squeezing the corresponding grip when the dot turned green. Only one alternative option was offered per trial and it was presented only once during the sequence. In 10% of trials, the alternative option was the *same* as the current option (i.e., the same reward size and the same remaining sequence length). In 20% of trials, the two dimensions of the choice were *congruent*: the alternative option had either a longer/same sequence length and a smaller/same reward size or a shorter/same sequence length and a larger/same reward. Note that the two options could not have the same reward size and sequence length. In the remaining 40% of trials, the two dimensions of the choice were *incongruent*: the alternative option either had a longer sequence length but a bigger reward size or a shorter sequence length but a smaller reward. The sequence length and reward size of the alternative option were drawn so that (i) if option A was offered as a current option and B as alternative on one trial, it was equally probable that A would be offered as an alternative and B as a current option on another trial, (ii) all sequence lengths (three to eight squeezes) were equally probable for the current and the alternative options, and (iii) before the choice, the numbers of squeezes performed were counterbalanced across sequences. As a consequence, starting with a long sequence was more probable (11 out of 18 trials) than a medium (5 out of 11 trials) and a short (2 out of 11 trials) sequence. All reward sizes and sides were equally probable.

### Training procedure

All three monkeys followed the same training procedure. They were first exposed (forced choice) to different sequence lengths, then had to choose between two options differing only in sequence length. Then, we exposed them to different reward sizes, and they had to choose between two options differing only in reward sizes. Finally, they had to choose between options differing both in reward sizes and sequence lengths. At the beginning of this training phase, both dimensions were favorable to the same option (congruent). We progressively increased the proportion of incongruent choices to 40%. Incongruent choices were more difficult for the monkeys since the reward size was greater for one option whereas the sequence length was shorter for the other option. We started recording their behavior and using pharmacological manipulations a least a month after they reached asymptotic performance on the final version of the task.

### Pharmacological procedure

We used three doses of clonidine—2.5, 5.0, and 7.5 μg/kg—which is a selective alpha-2 noradrenergic receptor agonist that, as well as potentially acting on post-synaptic alpha-2 receptors, previous studies have shown acts directly on LC neurons to suppress firing and consequently noradrenaline levels at the doses that we used (Kawahara et al. 1999; Fernández-Pastor et al. 2005). The doses that we used were below the doses that affected working memory functions in monkeys, which have been suggested to depend on the action of clonidine on post-synaptic alpha-2 receptors (Franowicz and Arnsten 1999). They were also below the sedative effect threshold determined in rats (Sara et al. 1995; Lapiz and Morilak 2006) and monkeys (Bouret and Richmond 2009), but elicited a subjective feeling of sedation humans (Jäkälä et al. 1999). Clonidine hydrochloride (C7897, Sigma-Aldrich, St. Louis, MO, USA) solutions were prepared freshly each day by dissolution in 1 mL saline for monkeys A and D and 0.5 mL for monkey E. The same volume of saline solution was given in saline condition. Drug or saline solution was injected intramuscularly 20 min before testing in monkeys’ home cages. Each dose or vehicle was given for five consecutive days (Monday to Friday). Order of drug and saline weeks (one drug week per dose and two saline weeks) was randomly assigned for each animal.

### Data analysis

Data were analyzed with Matlab software (MathWorks).

All sessions lasted 1 h. Monkeys’ overall willingness to work in the session was assessed by looking at all offered squeezes as long as monkeys were engaged in the task. Indeed, since monkeys tended to completely disengage before the end of the session, we removed these series of terminal rejected trials at the end of each session. The behavior during the sequence was examined by looking the %accepted squeezes during the completion of a trial. To look at the progression in the sequence effect, we looked only at trials where no choice was offered. The willingness to work on the first squeeze of a sequence was assessed in the same way but by taking all first choices since the subjects could not know whether a choice was going to be offered or not in a particular trial. Reaction time corresponds to the time between the display of the green dot and the crossing of the minimum force threshold for correct squeezes. Note that the green dot always appeared 2 s after the onset of visual cues indicating sequence length and reward size. Reaction time distributions for each grip and each monkey were log-transformed (to obtain Gaussian-like distributions) and z-scored (mean set to zero and variance to one).

To assess changes in the decision process, we looked at three variables: (i) the proportion of alternative options chosen per session, (ii) average chosen number of squeezes, (iii) the average chosen reward size, and (iv) the stability in choices. The stability is the mean probability to repeat the same choice (i.e., either taking the alternative or taking the current option) in each possible combination of differences in reward size (five possibilities − 2, − 1, 0, + 1, + 2) and sequence length (five possibilities − 4, − 2, 0, + 2, + 4) between the current and the alternative options. The stability is therefore maximal if monkeys are perfectly stable in their choices and minimal if they are completely random across all choices.

We also built a simple decision model where the value of each option (the current and the alternative) is computed as:

1 $$ V\left(\mathrm{option}\right)=R\left(\mathrm{option}\right)\hbox{--} {k}_{\mathrm{cost}-\mathrm{benefit}}.\kern0.5em SL\left(\mathrm{option}\right) $$

where *V*(option) is the value, *R*(option) the reward size, and *SL*(option) the sequence length (remaining number of squeezes to perform to obtain the reward) of the considered option. Both are properties of the option displayed on the screen and learned by subjects during training. The parameter *k*_cost-benefit_ represents the relative sensitivity to reward size and sequence length in the evaluation of the option. Hence, the higher this parameter, the higher the sensitivity to the cost (sequence length) compared to the benefit (reward size). A change in this parameter would represent a change in the evaluation of the options and more specifically in the way that the cost (sequence length) is weighted against the benefit (reward size). For example, an increase in this parameter means that subjects became more sensitive to the cost and were ready to sacrifice some reward in order to avoid having to perform squeezes. The values of the two options are compared to determine the probability to take the alternative option as follows:

2 $$ P(AO)=\frac{1}{1+\mathit{\exp}\left(-\left(V(AO)\hbox{--} V(CO)+\mathrm{bias}\right).\mathrm{consistency}\right)} $$

where *P*(*AO*) is the probability to take the alternative option and *V*(*AO*) and *V*(*CO*) the values of the alternative and current options respectively computed with Eq. 1. The bias parameter represents the tendency to either take the alternative option (positive value) or the current option (negative value). A change in this parameter would represent a change in the monkeys’ likelihood of switching or staying when presented with a choice such that subjects became more (decrease in bias) or less (increase in bias) reluctant to disengage from the option they are currently in. The consistency parameter represents the extent to which choices were consistent with the subjects’ subjective evaluation of the options. In other words, the smaller this parameter, the more likely a monkey deviates from selecting the option with the highest value. A change in this parameter can be understood as a change in how value-guided choices are. A greater consistency in choices corresponds to a more rigid way of making value-based decisions and a hampered ability to explore other options.The three parameters k_cost-benefit_, bias, and consistency in Eqs. 1 and 2 were estimated by inverting the model so as to minimize the free energy, using a variational Bayes approach under Laplace approximation (Friston et al. 2007; Daunizeau et al. 2009), implemented in a Matlab toolbox (Daunizeau et al. 2014). Whereas the model-based *consistency* parameter and model-agnostic *stability* measure capture similar aspects of the behavior, an increase of the consistency parameter would show that choices became more strongly influenced by the cost-benefit analysis, whereas an increase in the stability measure simply would show an increased likelihood of repeating a particular choice.

We extended the model-based analysis by including several parameters in the *full model*:

3 $$ P(AO)=\frac{1}{1+\mathit{\exp}\left(-\left(\begin{array}{c}V(AO)\hbox{--} {k}_{\mathrm{evaluation}}.V(CO)+\mathrm{bias}+\mathrm{side}\ \mathrm{bias}.\mathrm{side}\\ {}+{k}_{\mathrm{squeezes}\ \mathrm{done}}.\mathrm{squeezes}\ \mathrm{done}\end{array}\right).\mathrm{consistency}\right)} $$

where *side* is the side of the alternative option (+ 1 if on the right, − 1 if on the left) and *squeeze done* the number of squeezes performed to get to the choice. The six parameters *k*_cost-benefit_ (Eq. 1), bias, side bias, *k*_squeezes done_, and consistency in Eqs. 1 and 3 were estimated as above.

Reaction time corresponds to the time between the display of the green dot and the crossing of the minimum force threshold for correct squeezes. Reaction time distributions for each grip and each monkey were log-transformed and z-scored (mean set to zero and variance to one). We compared reaction times across different squeeze types. When comparing *choice* and *no choice* reaction times, no choice reaction time corresponds to trials where no choice was offered but in principle could have been (Fig. 1b) and choice reaction time corresponds to points in the sequence where monkeys were presented with an alternative option but stayed with the original option. To evaluate the effect of choice difficulty on reaction time, we examined the effect of the absolute difference in value between two options, since choices get more difficult when this difference decreases. We binned the data according to the absolute difference in value of the options (five bins, same as used to plot the Fig. 4d, e) and took only choices where monkeys stayed with the current option and made a correct decision.

Motivational changes with treatment were assessed by two variables: force peak and willingness to work.

To calculate force peak, force time series for both grips were low-pass filtered at 15 Hz (zero-phase second-order Butterworth filter) and we took the maximal value of the force signal between two crossings of the minimal force threshold.

Willingness to work was calculated based on three separate measures. The first corresponds to the proportion of accepted squeezes per session during the 1-h session (including the time at the end of the session when monkeys disengaged in the task). The second examines willingness to work specifically at the beginning of each sequence (i.e., the first squeeze of all trials). The third measure estimated willingness to work *across* sequences of given length and reward size by taking all trials where no choice was offered in a given drug condition for each monkey and fitted using the following model:

4 $$ \%\mathrm{correct}\ \mathrm{squeezes}(n)=\left(1\hbox{--} {k}_{\mathrm{intercept}}.\mathit{\exp}\left(-{k}_{\mathrm{slope}}.n\right)\right).100 $$

where *n* is the number of squeezes done in the sequence. Parameters *k*_intercept_ and *k*_slope_ were estimated for each dose and each monkey using the same procedure as for the parameters of Eqs. 1 and 2.

### Statistical analysis

Data are plotted as mean ± standard error to the mean. Statistics used are indicated in the “Results” section. Comparisons between means were performed using parametric tests (ANOVA and *T* test). At the group and the subject level, we performed multi-level linear regressions on z-scored distributions (reaction times and force peaks) using the function glmfit in Matlab. In cases when distributions were not z-scored, we took into account the variability in mean across subjects by fitting an intercept for each subject using the function fitlme in Matlab. This step is equivalent to subtracting the mean in the data for each subject. The general equation was:

5 $$ y={\beta}_0+{\beta}_0\left(\mathrm{subject}\right)+{\sum}_{\mathrm{i}}{\beta}_{\mathrm{i}}.{x}_{\mathrm{i}} $$

where *y* is the data, *β*_0_ a constant, *β*_0_(subject) a constant fitted for each subject, *x*_i_ the experimental factors, and *β*_i_ their weights in the linear regression. *T* tests were performed on distributions of the linear regression weights. In all cases, *t* values and degrees of freedom reflect the fact that we looked at the behavior across four doses for three subjects (12 data points for each condition) and fitted at least an intercept (*β*_0_) and a slope for the linear effect of the drug, which gives 10 degrees of freedom if there is only one condition. All statistical tests were two-sided. *p* > 0.05 was considered to be not statistically significant. We evaluated the quality of our models’ fit using balanced accuracy (between 0 and 1) computed as (Brodersen et al. 2010):

6 $$ \mathrm{Balanced}\ \mathrm{accuracy}=\frac{1}{2}.\left(\frac{\mathrm{true}\ \mathrm{positives}}{\mathrm{true}\ \mathrm{positives}+\mathrm{false}\ \mathrm{negatives}}+\frac{\mathrm{true}\ \mathrm{negatives}}{\mathrm{true}\ \mathrm{negatives}+\mathrm{false}\ \mathrm{positives}}\right) $$

## Results

### Overview of the task and subjects’ performance

We trained three monkeys to perform the task depicted in Fig. 1. In each trial, monkeys performed series of actions (squeezing a grip six, eight, or ten times) to get a small, medium, or large fluid reward (Fig. 1a). Note that the amount of force required to complete the trial was minimal and monkeys always succeeded to complete a squeeze when they tried. In 70% of trials, we introduced a choice by presenting an alternative option before monkeys could complete the trial. This alternative option, presented on the opposite side of the monitor compared to the current option, was also characterized by a given number of squeezes and a given reward size. Thus, monkeys could either choose to continue with the current option by squeezing the same grip as before, or switch to the other grip to start completing the alternative option and obtain the corresponding reward.

Monkeys performed on average (across treatment conditions) 61, 71, and 55 trials per session (monkeys A, D, and E respectively). Note that since the amount of force required to complete the squeeze was minimal, monkeys always succeeded to complete it when they tried. Thus, a failure to squeeze the bar was always interpreted as a rejection of the current offer, either at the onset or in the middle of a sequence. Overall, when monkeys were performing the task, they engaged in respectively 90.4, 95.8, and 78.9% of the offered forced choice squeezes (all “no choice” squeezes included) (monkeys A, D, and E respectively, mean across treatment conditions, no significant effect of treatment condition: linear effect taking into account the variability across subjects, *t*(10) = − 0.52, *p* = 0.61). Note that these relatively low percentages are due to the fact that the first squeezes of the sequence were often refused. On the choice squeezes, they were also very unlikely to disengage, and they accepted 99.4, 99.6, and 99.8% of choices (monkeys A, D, and E respectively, the same as above *t*(10) = − 0.02, *p* = 0.79).

### Behavior under saline

**Fig. 2:**
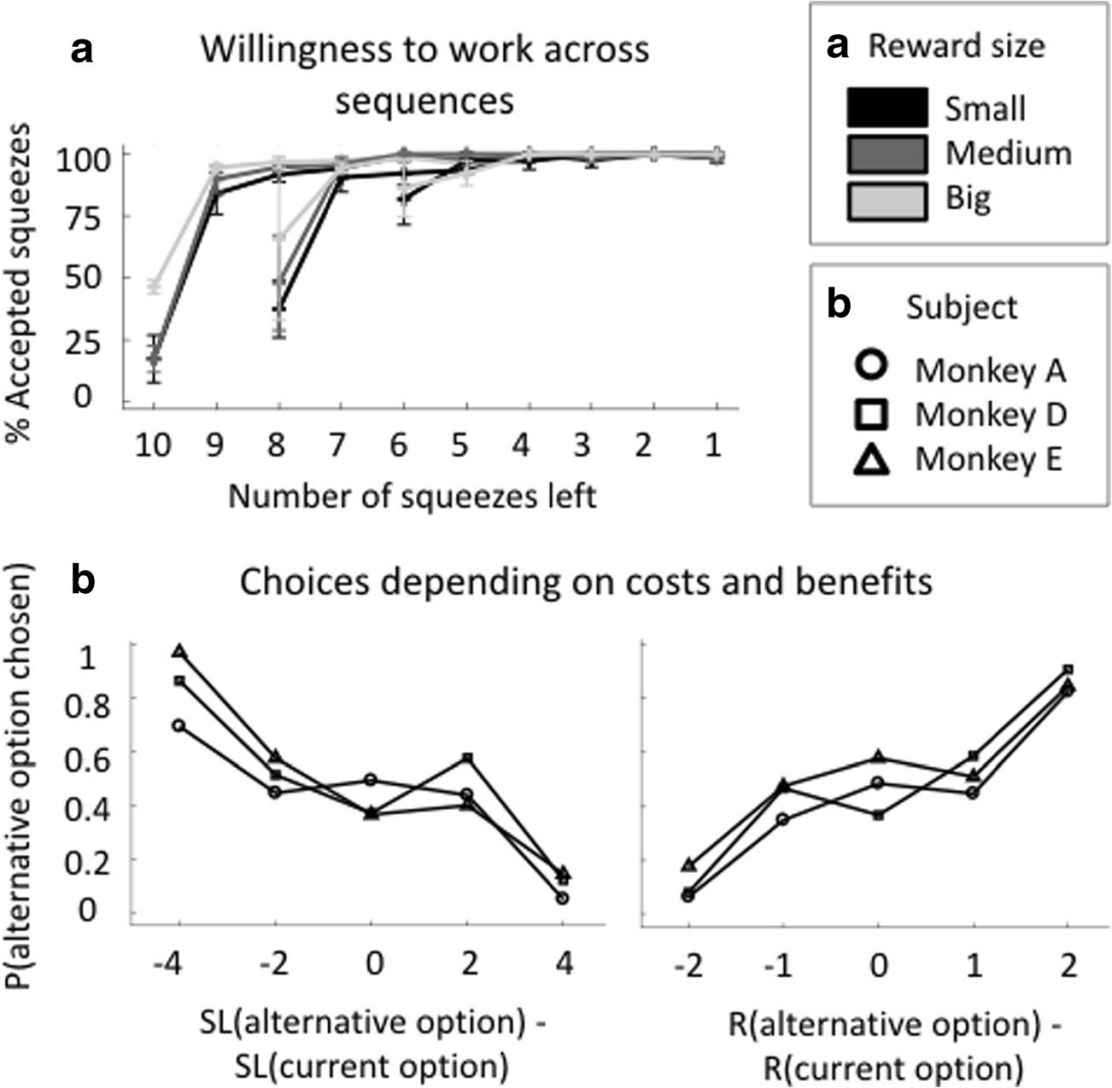
Behavior under saline. **a** Willingness to work across the sequence. Number of correct squeezes/total number of squeezes depending on the number of remaining squeezes to complete the sequence in trials where no choice was offered (in %). Color code corresponds to reward size and sequences of different sequence length have different starting point. Mean across monkeys, error bars represent standard errors to the mean. **b** Choices depending on costs and benefits. Probability to take the alternative option according the difference in sequence length (left) and reward size (right) of the options. Mean over all saline session for each subject. Symbols correspond to each subject (circle: monkey A, square: monkey D, triangle: monkey E)

We first examined monkeys’ behavior under saline. During the sequence, monkeys’ acceptance rate increased sharply after the first squeeze: monkeys sometimes rejected the offer at the beginning of a sequence but virtually never gave up in subsequent steps (Fig. 2a). Their acceptance rate was positively modulated by the reward size (linear effect taking into account the variability across subject on all first squeeze, *β* = 0.27 ± 0.12, *t*(24) = 2.15, *p* = 0.04) and negatively modulated by the sequence length (*β* = − 0.71 ± 0.13, *t*(24) = − 5.67, *p* < 0.001). We also performed a logistic regression on the first squeeze of each sequence for each individual monkey, and both effects were also significant at the subject level except for the reward size effect on monkey D which was only marginally significant (monkey A: *β*(sequence length) = − 0.82 ± 0.05, *t*(2740) = −15.83, *p* < 0.001, *β*(reward size) = 0.89 ± 0.05, *t*(2740) = 13.09, *p* < 0.001; monkey D: *β*(sequence length) = − 0.12 ± 0.05, *t*(1553) = − 2.41, *p* = 0.02, *β*(reward size) = − 0.09 ± 0.05, *t*(1553) = − 1.83, *p* = 0.07; monkey E: *β*(sequence length) = − 1.21 ± 0.10, *t*(1491) = − 11.78, *p* < 0.001, *β*(reward size) = 0.83 ± 0.07, *t*(1491) = 12.66, *p* < 0.001). We also examined the influence of task factors on monkeys’ reaction times to the green dot (go signal). By contrast with the acceptance rate, reaction times at the group level did not show a significant modulation by either sequence length (multi-level linear regression on all successful first squeezes, *t*(24) = 1.71, *p* = 0.10) or reward size (*p* = 0.69). At the subject level, only monkey A showed a significant effect of sequence length (*β*(sequence length) = 0.10 ± 0.03, *t*(1042) = 3.28, *p* = 0.001) and the effect of reward size approached significance (*t*(1042) = −1.82, *p* = 0.07) (all others: *p* > 0.24). Overall, monkeys’ reaction times were not modulated by the experimental factors, whereas their willingness to work on the first trial was.

**Fig. 3:**
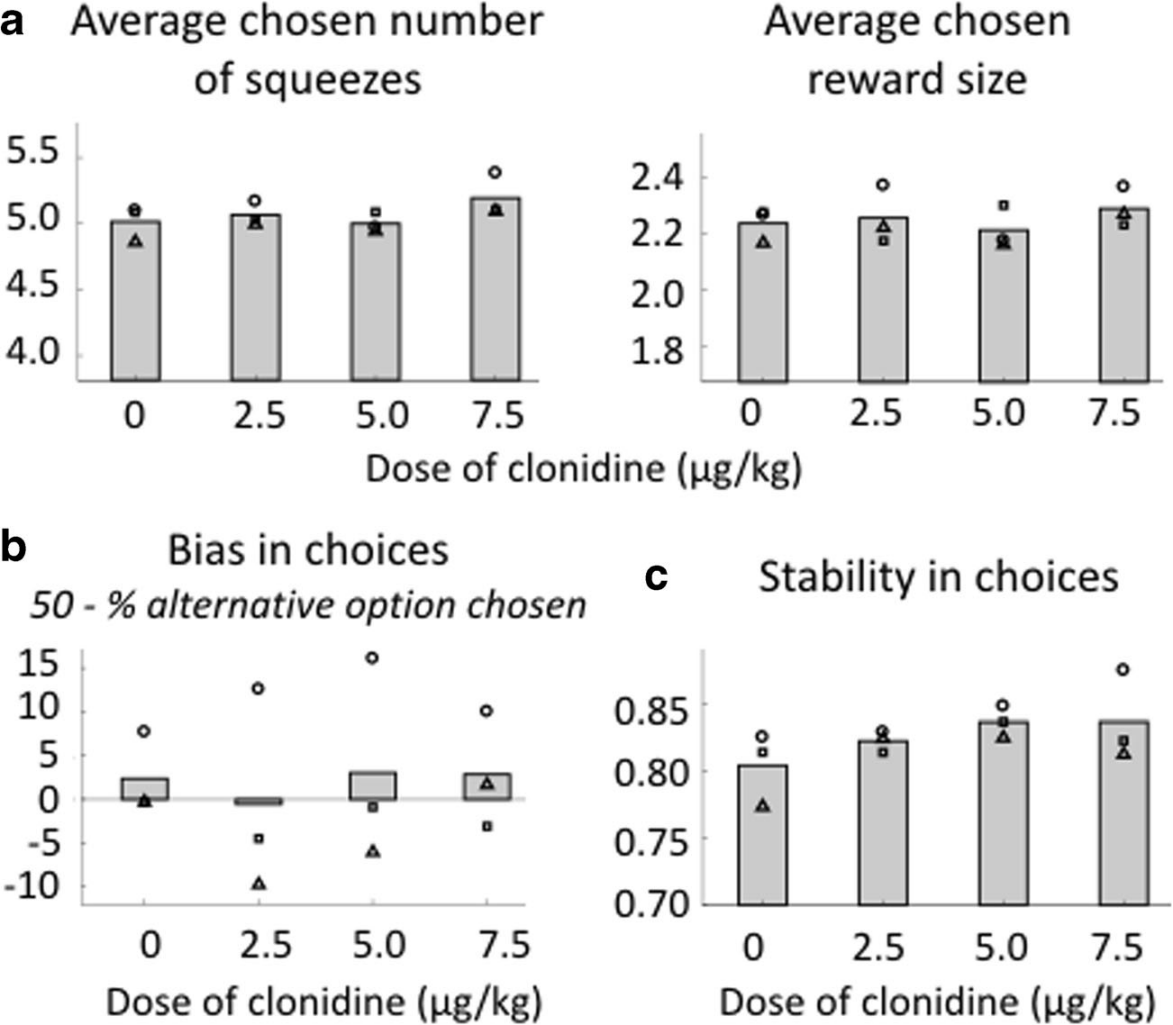
Effect of clonidine on decision-making. **a** Average chosen number of squeezes and reward size. Average number of squeezes chosen to be performed at the choice point in each treatment condition. There was no effect of treatment condition. Size of average reward chosen (1 for small, 2 for medium, and 3 for big) in each treatment condition. The same as **a**. There was no effect of treatment condition. Symbols correspond to each subject (circle: monkey A, square: monkey D, triangle: monkey E). There was no significant bias toward staying or switching across all treatment conditions. **b** Overall bias for alternative or current option. 50—mean across monkeys of the percentage of alternative option chosen for each treatment condition. The same as **a**. **c** Choices stability. Mean across monkeys and treatment condition. The same as **a**. Linear regression taking into account the variability across individuals revealed a significant positive linear effect of treatment condition (*P* < 0.05)

As expected, the monkeys’ choices between the current and the alternative options were affected both by the expected costs (number of remaining squeezes) and benefits (reward size) (Fig. 2b). The effects were significant both at the group level (multi-level linear regression taking into account the variability across subject: *β*(sequence length difference) = − 0.003 ± 0.0002, *t*(69) = − 12.70, *p* =< 0.001, *β*(reward size) = 0.003 ± 0.0002, *t*(69) = 12.29, *p* < 0.001) and at the subject level (multi-level linear regression on each subjects’ choices: monkey A: *β*(sequence length difference) = − 0.003 ± 0.0003, *t*(638) = − 11.52, *p* < 0.001, *β*(reward size) = 0.003 ± 0.0003, *t*(638) = 11.67, *P* < 0.001; monkey D: *β*(sequence length difference) = − 0.004 ± 0.0004, *t*(558) = − 9.49, *p* < 0.001, *β*(reward size) = 0.004 ± 0.0004, *t*(558) = 9.86,*p* < 0.001; monkey E: *β*(sequence length difference) = − 0.004 ± 0.0005, *t*(282) = − 7.43, *p* < 0.001, *β*(reward size) = 0.003 ± 0.0005, *t*(282) = 7.05, *p* < 0.001). Thus, all monkeys had clearly understood the quantities at stake for the choice, and they were affected in a similar manner by the two factors. Note that monkeys A and D had a significant bias for the current option and the alternative option respectively (monkey A: *β* = − 0.0007 ± 0.0001, *t*(558) = 2.15, *p* < 0.001; monkey D: *β* = 0.0003 ± 0.0001, *t*(638) = − 5.81, *p* < 0.001) whereas monkey E was not biased (monkey E: *t*(282) = 0.80, *p* = 0.42) (Fig. 3b).

This analysis of monkeys’ behavior under saline showed that their performance in the task was similar. However, we could observe different profiles. Monkey A had a general bias toward staying with the current option. The other male (monkey D) had the opposite bias, and the female (monkey E) had no bias. These idiosyncratic features of the monkeys’ behavior were taken into account in all our subsequent analyses. Indeed, as for the other behavioral measures (force, reaction times, stability in choices) and parameter estimates, we fitted a different intercept for each monkey.

### Effects of clonidine on behavioral flexibility: choices

We first measured the influence of clonidine on choices. We first looked at the average number of squeezes chosen and the reward size chosen (Fig. 3a), no matter whether it was a stay or switch choice, which provides a global estimate of the animals’ relative sensitivity to costs and benefits. This measure was also not reliably affected by the treatment (linear regression taking into account subject variability, *t*(10) = 1.85, *p* = 0.09 and *t*(10) = 0.65, *P* = 0.53, for squeezes and reward size respectively).

We next examined the influence of clonidine on the choice bias toward the current versus alternative option, over and above the influence of expected costs (number of squeezes) and benefits (reward sizes). Because all options were offered in equal proportions as current and alternative options, any departure of the probability to take the alternative option from 50% would represent a bias toward staying or switching. At the group level, there was no significant bias toward staying or switching across all treatment conditions (linear regression taking into account subject variability, *t*(10) = 0.52, *p* = 0.52), and the bias was not different from zero in any condition (*T* test, *p* > 0.47, for all doses) (Fig. 3b).

We then looked the stability in choices across doses. As shown in Fig. 3c, there was a clear linear increase in stability across doses of clonidine, which means that with increasing doses of clonidine, the monkeys became increasingly likely to make the *same* decisions when faced with the *same* type of choice (linear regression taking into account variability across subjects, *β* = 0.559 ± 0.183, *t*(10) = 3.06, *p* = 0.01).

To capture the specific influence of clonidine on distinct components of decision-making, we built a simple choice model depicted in Eqs. 1 and 2 in “Material and methods”. In this model, the value of each option corresponds to a trade-off between reward at stake and sequence length, controlled by a parameter *k*_cost-benefit_. The probability to select a given option depends on (i) the value difference with the alternative and (ii) a fixed bias, e.g., a preference for either staying with the current option or taking the alternative, as well as (iii) the choice consistency, which determines the degree to which choices are consistent with the evaluation.

**Fig. 4:**
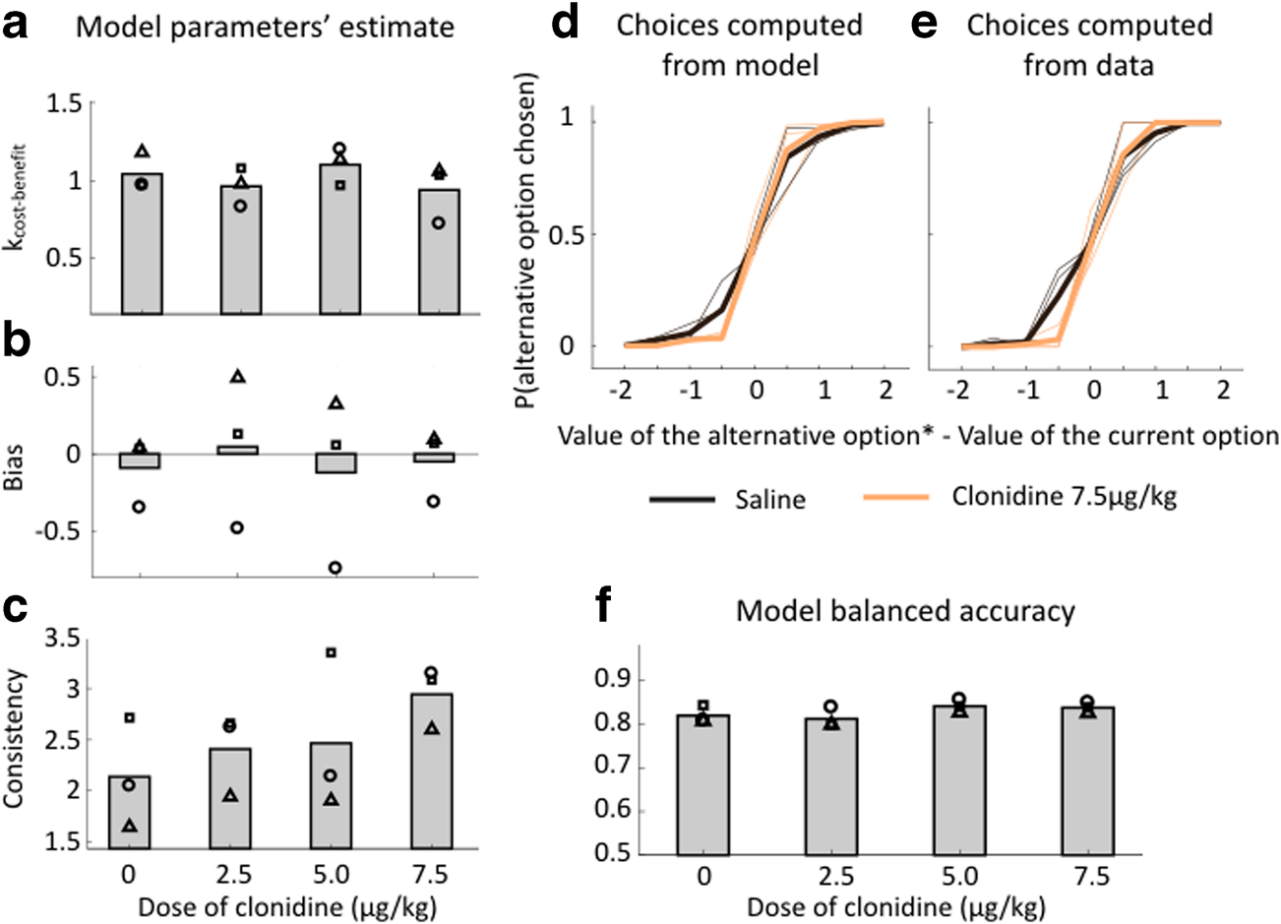
Clonidine specifically affects consistency in choice. Mode-based analysis: parameters’ estimate. **a** *k*_cost-benefit_ parameter estimates. Mean across monkeys of the *k*_cost-benefit_ parameter estimates for each treatment condition in the simple choice model. Symbols correspond to each subject (circle: monkey A, square: monkey D, triangle: monkey E). There was no effect of treatment condition at the group level. **b** Bias parameter estimates. The same as **a**. There was no effect of treatment condition at the group level. **c** The same as **a**. Multi-level regression on estimated beta parameters taking into account the variability across subjects revealed a significant effect of treatment condition (*P* < 0.01). Probability to take the alternative option. Probability to take the alternative option depending on the value of the current option and the corrected value of the alternative option (V(alternative option)* = V(alternative option) + bias). Thin lines are subjects’ curves and thick lines are the mean across monkeys. Color code corresponds to either saline or the highest dose of clonidine. **d** Choices computed from model. Probability to take the alternative option computed with the choice model (estimates of the consistency parameter). **e** Choices computed from data. The same as **d** but the probability to take the alternative option is computed with subjects’ actual choices for the estimated values. **f** Model balanced accuracy. The same as **a**. There was no effect of treatment condition at the group level

As shown in Fig. 4, we looked at the effect of the treatment on the three parameters of the choice model (*k*_cost-benefit_, bias, and consistency) (see online resource ESM_1 for the subjects’ parameters’ estimate). The parameter *k*_cost-benefit_ describing the relative sensitivity to reward and sequence length was significantly different from zero, indicating that monkeys readily integrated these two factors to guide their behavior (all *p* < 0.01). Had either the sensitivity to sequence length or reward size changed following administration of clonidine, this parameter would have varied. For example, an increase in effort sensitivity would have been translated in an increase in the *k*_cost-benefit_ parameter because animals would have given up some reward to exert fewer squeezes. But as shown in Fig. 4a, this parameter estimate was again not affected by the treatment (linear regression taking into account variability across subjects, *t*(10) = − 0.10, *p* = 0.92), indicating a lack of effect of clonidine on the cost-benefit analysis. In line with the previously described model-agnostic analysis (Fig. 3b), monkeys had different bias parameter values, but there was no systematic bias to stay with the current option or switch to the alternative at the group level (bias parameter not significantly different from zero, all *p* > 0.55) and no effect of treatment on this bias parameter (linear regression taking into account variability across subjects, *t*(10) = − 0.10, *p* = 0.92) (Fig. 4b). By contrast, clonidine induced a dose dependent increase in choice consistency (Fig. 4c). To analyze this formally, we ran a linear regression taking into account the variability across subjects. This revealed a significant linear effect of dose on the consistency parameter’s estimates (*β* = 0.248 ± 0.070, *t*(10) = 3.54, *p* < 0.01). Figure 4d, e illustrates the influence of the highest dose of clonidine on choices. The slope of the choice curve is noticeably higher under clonidine, reflecting a reliable increase in choice consistency (see online resource ESM_2 for individual subject’s choice curves).

To ensure that our model accurately captured monkeys’ choices, we computed the model balanced accuracy for each subject and each treatment condition and it was overall between 0.80 and 0.86 (Fig. 4f). There was close but not significant positive effect of dose on balanced accuracy (linear regression taking into account variability across subjects, *β* = 0.008 ± 0.004, *t*(10) = 2.08, *p* = 0.06) and close but not significant correlation between the balanced accuracy and the consistency parameter (linear regression taking into account variability across subjects, *β* = 12.39 ± 6.13, *t*(10) = 2.02, *p* = 0.07). However, because these statistical tests were close to the significance threshold and to ensure that the balanced accuracy was not driving the effect on the consistency parameter, we compared a model in which the consistency parameter is linearly dependent on the balanced accuracy (linear regression taking into account variability across subjects, Bayesian information criterion: BIC = 23.9) and linearly dependent on the dose (same, BIC = 18.8). Since the later model won the comparison (∆BIC = 5.1 > 3, meaning that there is a strong evidence in favor of the dose-effect model), the dose effect was better explained by a change on the consistency parameter than by a change in goodness of fit, evaluated by the balanced accuracy.

Finally, we used a more complex model (*full model*) to try to capture other factors potentially affecting choices. Notably, we generally found a significant side bias in monkeys’ choices but it was not systematically significant and changed direction across treatment conditions (or weeks of testing) in the same animal. We also added a parameter capturing the effect of the number of squeezes done before the choice and found a significant positive effect of this parameter on the probability to take the alternative option. Hence, monkeys were more likely to take the alternative option if they had done more squeezes to get to the choice. We also added a parameter to capture an imbalance in the evaluation of the options, but this parameter was not different from 1 across all subjects and doses (*T* test, *p* = 0.08). Overall, including these parameters in the choice model did not affect the results presented above, and there was no effect of the treatment on any of them (linear regression taking into account the variability across subjects: all *P* > 0.41), except on the consistency parameter (*β*(consistency) = 0.265 ± 0.088, *t*(10) = 3.00, *P* = 0.01) consistency (see online resource ESM_3 for subjects’ full model parameters estimates).

### Effect of clonidine on reaction times

**Fig. 5:**
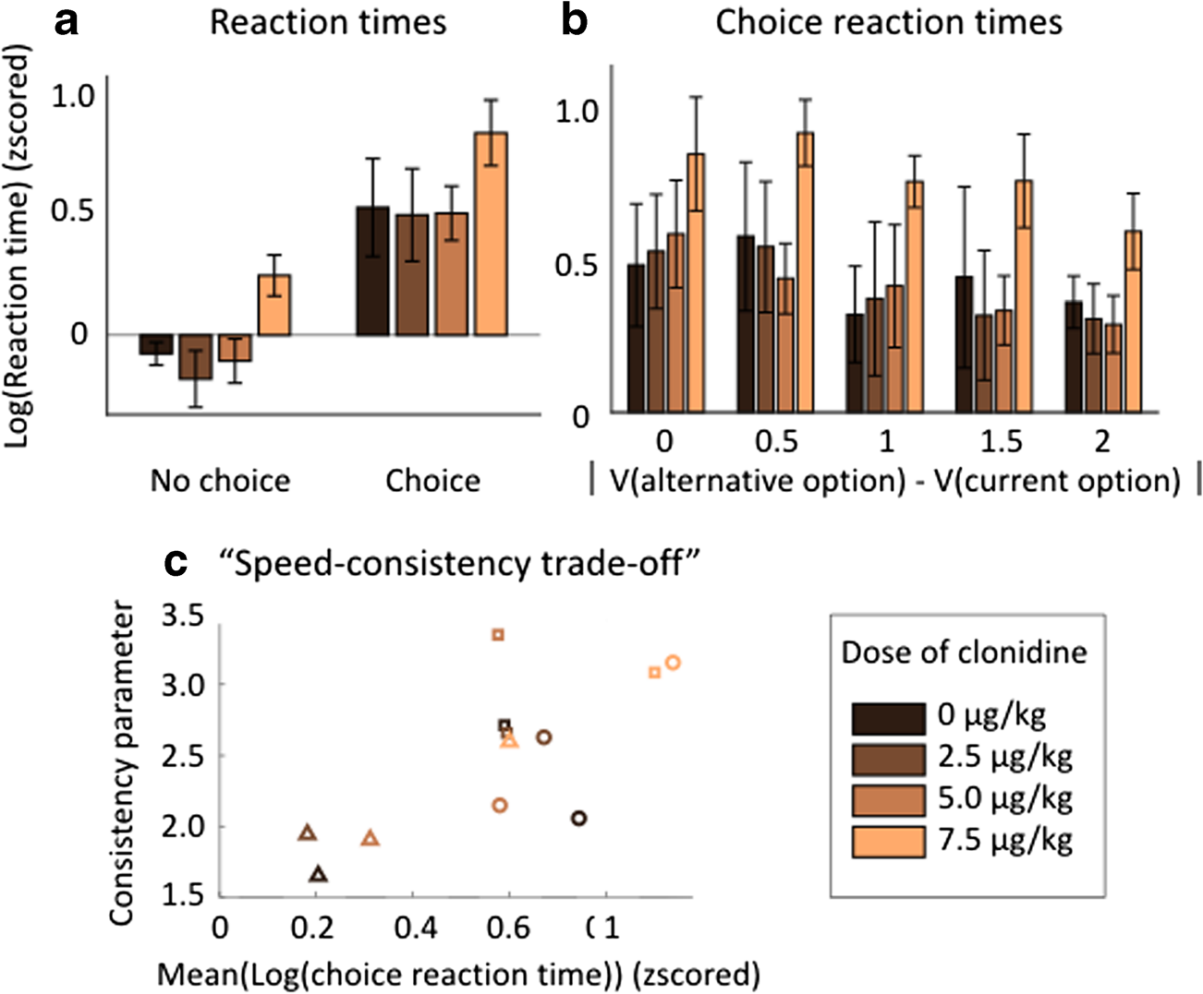
Effects of clonidine on reaction times. **a** Reaction times for no choice and choice. Reaction times in two squeeze types (no choice and choice) for each treatment condition. No choice squeezes are matched to choice squeeze for position in the sequence and compared to choices in which subject did not change grip. Reaction time distributions for each grip and each monkey are logged z-scored (mean sets to zero and variance to one). Mean across monkeys, error bars represent standard errors to the mean. Color code corresponds to treatment condition. Linear regression on log-transformed reaction times revealed a significant positive linear effect of choice (*P* < 0.001) and treatment condition (*P* < 0.01). **b** Choice reaction times across differences in options’ value. Reaction times across five bins of the absolute difference in value of the two options (choice difficulty) for correct current option choices only. The same as **a**. Linear regression on log-transformed reaction times revealed a significant negative linear effect of choice (*P* = 0.008) and treatment condition (*P* < 0.001) but no interaction. **c** Correlation between choice reaction times and consistency parameter estimates. Reaction times correspond to the time between the display of the green dot and the crossing of the minimum force threshold for correct squeezes where monkeys had to make a choice and did not change grip. The consistency parameter was computed by fitting the choice model. They were computed for each subject and treatment condition. The same as **a**. The correlation between these two parameters was significant (*P* < 0.01)

We next evaluated the effects of clonidine on reaction times across task conditions. We separated squeezes where monkeys had to make a choice between two options from equivalent points in the sequence on single option squeeze, where they only squeezed the grip to progress through the trial. As classically observed, monkeys were slower to respond in choice than no-choice trials (Fig. 5a). We examined the influence of clonidine on reaction times in these two types of trials, and a multi-level linear regression taking into account variability across subjects revealed a significant linear effect of choice (*β* = 0.641 ± 0.022, *t*(21) = 6.30, *p* < 0.001) and dose (*β* = 0.070 ± 0.007, *t*(21) = 2.90, *p* < 0.01), but no significant interaction (*t*(20) = 0.13, *p* = 0.89). Hence, clonidine significantly increased reaction times, but its effects were undistinguishable between choice and non-choice conditions. We also separated choice reaction times according to the absolute difference in value of the two options (choice difficulty) and found a negative main effect of difference in value on reaction times (multi-level linear regression, *β* = − 0.31 ± 0.11, *t*(57) = − 2.73, *p* = 0.008) and a main effect of dose (*β* = 0.41 ± 0.11, *t*(57) = 3.63, *p* < 0.001) but once again no interaction (*t*(56) = − 0.45, *p* = 0.65) (Fig. 5b). Hence, both clonidine and choice difficulty increase reaction time but their effects are simply additive, indicating that clonidine does not interfere with the influence of difficulty on reaction times. Overall, monkeys’ reaction times were clearly modulated across conditions: animals took longer to respond when they had to make a choice, especially if it was difficult. High doses of clonidine also increased monkeys’ reaction times, but because their effects were equivalent across conditions (no interaction), it did not affect the influence of difficulty on reaction times.

Together, our analyses therefore revealed two effects of clonidine on behavior: it dose-dependently increased both choice consistency (as captured by the model-based analysis) and choice reaction times. We then examined the relation between these two effects across treatments and animals. We found a positive correlation (linear regression taking into account variability across subjects, *β* = 0.321 ± 0.095, *t*(10) = 3.47, *p* < 0.01) between the estimated consistency parameter and the choice reaction time (Fig. 5c). This correlation between the effect of treatments on reaction time and choice consistency suggests that clonidine affects a single functional entity, which we will refer as the “speed-consistency trade-off.”

### Effect of clonidine on motivation: willingness to work

**Fig. 6:**
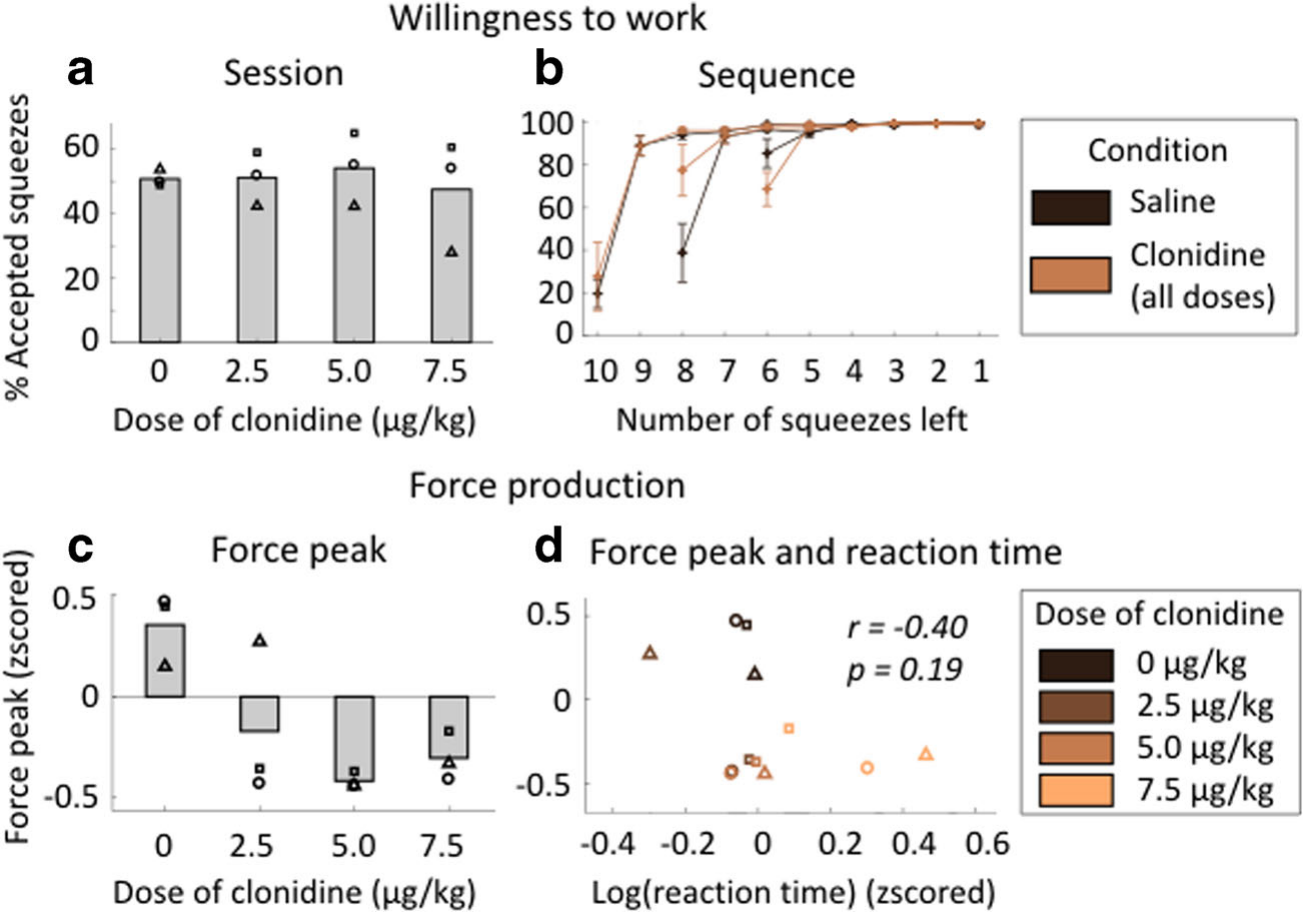
Effect of clonidine on motivation. Willingness to work. **a** Willingness to work during the session. Number of accepted squeezes/total number of squeeze during the session (1 h) for each treatment condition (in %). Mean across monkeys. Symbols correspond to each subject (circle: monkey A, square: monkey D, triangle: monkey E). There was no significant effect of treatment condition. **b** Willingness to work across the sequence. Number of correct squeezes/total number of squeezes depending on the number of remaining squeezes to complete the sequence in trials where no choice was offered (in %). For simplicity, only the effect of treatment (color code: saline vs. clonidine, all doses pooled) and sequence length are shown here. Mean across monkeys, error bars represent standard errors to the mean. Force production. **c** Force peak. Maximal value of the force signal between two crossings of the minimal force threshold for correct squeezes. Force peak distributions for each grip and each monkey are z-scored (mean sets to zero and variance to one). Mean across monkeys. The same as **a**. Linear regression showed a negative effect of treatment condition on peak force (*P* < 0.01). **d** Force peak and reaction time.  Force peak is the same as in **c**. Reaction time corresponds to the time between the display of the green dot and the crossing of the minimum force threshold for correct squeezes for each treatment condition. Reaction time distributions for each grip and each monkey are logged and z-scored (mean sets to zero and variance to one). Symbols correspond to each subject (the same as **a**). Color code corresponds to treatment condition

After assessing the implication of noradrenaline in behavioral flexibility, we examined the influence of clonidine on two additional behavioral measures that are classically used to assess motivation: willingness to work and physical force production (Fig. 6). We first measured monkeys’ willingness to work by counting the proportion of accepted squeezes. Since the action is very easy, the number of squeezes that they perform in a session reflects their general motivation to engage with the task. At the subject level, we found a marginally significant effect of the dose on the willingness to work during 1-h-long sessions for monkey E only (monkey E: *β* = − 0.08 ± 0.02, *t*(2) = − 4.14, *p* = 0.054; monkey A: *β* = 0.02 ± 0.01, *t*(2) = 2.53, *p* = 0.15; monkey D: *β* = 0.04 ± 0.02, *t*(2) = 1.77 *p* = 0.22). However, it was not significantly affected by dose at the group level (linear regression taking into account the variability across subjects, *t*(10) = − 0.36, *p* = 0.73) (Fig. 6a).

We next examined the effect of clonidine on animals’ trial-by-trial willingness to work trial as a function of the upcoming effort cost and future reward size. To do this, we first examined willingness to work on the first squeeze of all sequences. We included every trial, since there was no way for monkeys to predict at the start of a sequence if a choice was going to be offered later in that sequence. We again examined the influence of reward size and sequence length on the willingness to perform the first squeeze using a linear regression taking into account the variability across subjects. This analysis revealed a significant negative effect of sequence length (i.e., the animals were less willing to engage on long sequences: *β* = − 18.81 ± 2.44, *t*(104) = − 7.71, *p* < 0.001) and a marginally significant positive effect of reward size (animals were more willing to engage for greater reward: *β* = − 4.79 ± 2.44, *t*(104) = 1.96, *P* = 0.052), but no effect of dose of clonidine (*t*(104) = 1.43, *P* = 0.16) and no interaction with sequence length (*t*(102) = 0.20, *p* = 0.84) or reward (*t*(102) = − 0.65, *p* = 0.52). We also looked at the effect of clonidine on reaction times during the first squeeze of the sequence and found a positive effect of sequence length (multi-level linear regression, *β* = 0.08 ± 0.04, *t*(104) = 2.05, *p* = 0.04) but no effect of reward size (*t*(104) = 0.08, *p* = 0.93). We found a positive main effect of dose (*β* = 0.09 ± 0.03, *t*(104) = 2.93, *p* = 0.004) but no interaction with sequence length and reward size (both *p* > 0.40). Hence, the treatment did not interfere with subjects’ evaluation of whether or not to engage in the sequence.

We then examined the influence of clonidine on adjustments to willingness to work across the steps of a trial (Fig. 6b). In all treatment conditions, monkeys displayed a sharp increase in their willingness to work after the first squeeze. We fitted the curves depicted in Fig. 6b with Eq. 4 for all trials during which no choice was offered. In this model, *k*_intercept_ controls the intercept (initial willingness to work) and *k*_slope_ the slope of the rise of the willingness to work across the sequence. We then examined the influence of reward and sequence length on each of these parameter estimates using a multi-level linear regression taking into account the variability across monkeys. The parameter *k*_intercept_ displayed a significant positive linear effect of sequence length (*β* = 0.160 ± 0.019, *t*(104) = 8.09, *p* < 0.001) and a negative effect of reward size (*β* = − 0.048 ± 0.019, *t*(104) = − 2.44, *p* < 0.05). Importantly, however, there was again no significant linear effect of dose (*t*(104) = − 1.85, *p* = 0.07) and no interaction with either the sequence length (*t*(102) = 0.32, *p* = 0.75) or the reward size (*t*(102) = − 0.12, *p* = 0.91). Moreover, neither the task factors (reward and sequence length) nor the dose of clonidine and its interaction with the tasks factors affected the parameter *k*_slope_, which captured the slope of the change in willingness to work across the sequence (all *p* > 0.30).

In short, while monkeys’ willingness to start and persist with an action sequence was sensitive to the sequence length and reward size on each trial, none of these parameters were affected by clonidine.

### Effect of clonidine on motivation: force production

Lastly, we examined the effect of clonidine on another key component of motivation: force production. As shown in Fig. 6c, force peak was significantly decreased under clonidine treatment (linear regression, *t*(10) = − 3.24, *p* < 0.01).

As described in an earlier section, clonidine also increased overall the reaction times (linear regression, *t*(10) = 2.63, *P* = 0.02). Therefore, we considered the possibility that clonidine was having non-specific motivational effect, for instance on arousal or vigilance, which would then be responsible for both longer reaction times and smaller force peaks. In such a scenario, we might therefore expect a strong relation between the effects of clonidine on force peak and reaction time.

To assess whether the effect of dose was the same on the two measures, we performed a two-way ANOVA on z-scored force peak and reaction as the dependent variables. Independent variables were dose and measure type (force peak vs. reaction time). We found a significant main effect of dose (*F*_3,16_ = 5.25, *P* = 0.01) and measure (*F*_1,16_ = 4.72, *p* = 0.04), but importantly also a significant interaction between the two (*F*_3,16_ = 8.59, *p* = 0.001), indicating that the effect of clonidine differs significantly between the two measures. We further explored this using a linear regression (Fig. 6d); there was no reliable correlation between the two measures (*r*(10) = − 0.4, *p* = 0.19). Moreover, we ran separate linear regressions excluding the highest dose of clonidine, where the effect on reaction times appeared most prominently (see Fig. 5a). While the effect on force peak was still significant even when only analyzing the low and mid doses (*t*(7) = − 3.93, *p* = 0.005), this was not the case for reaction time (*t*(7) = 0.16, *p* = 0.88). This was coherent with a stronger linear effect of dose on force peak (*β* = − 0.222 ± 0.068) than on reaction time (*β* = 0.106 ± 0.040). Therefore, effort production appears more sensitive to clonidine manipulations than any reaction time measures.

Finally, given that we had found a relationship between changes in reaction times and choice consistency under different doses of clonidine, we explored whether there was any similar connection between each animals’ average force peak and consistency parameter. However, we found no significant linear relationship between measures (*t*(10) = − 1.39, *p* = 0.15). Together, this demonstrates that clonidine has a specific, separable, and dose-dependent influence on different aspects of motivation and that the effect of clonidine on force production and choice behavior (as indexed by the monkeys’ speed-consistency trade-off) is at least partially dissociable.

## Discussion

We used a novel decision-making task, in which monkeys make sequential actions for reward and, on most trials, choose whether to stay with their current sequence or to switch to an alternative based on the costs and benefits of the options. Using systemic injections of clonidine, at doses known to decrease LC activity (Kawahara et al. 1999; Fernández-Pastor et al. 2005), we showed that noradrenaline was causally involved in controlling both the variability in choices (which can be interpreted as a simple form of behavioral flexibility) and the response speed. We also showed that noradrenaline was involved in regulating the intensity of the force produced, in line with a potential role in promoting effortful actions. Importantly, the effects on force were distinct to those on response speed, and both measures were dissociable from animals’ willingness to work, which remained unaffected at all doses of clonidine. Similarly, the dose-dependent increase in choice consistency under clonidine occurred in the absence of any changes in the rate of switching choices. These data therefore confirm, refine, and potentially reconcile recent theories of implicating noradrenaline in a behavioral flexibility and motivation, beyond its known general influence on vigilance and arousal.

Overall, we found a coherent effect of the treatment across the three subjects. For instance, in spite of a difference in their initial bias for the current versus the alternative option, we found that the treatment had no effect on this aspect of the behavior. The only potential difference was that we only found a marginally significant effect of the treatment in monkey E. This could be due to a subtle difference between the effect of treatment in monkey E and the others. Indeed, monkey E is a small young female (as opposed to two heavy adult males), and it is possible that the pharmacokinetic of the drug was different in this animal. Critically, however, she shows the same effect of the drug on our main measures of interest (reaction time, force, and choice consistency) as the two other animals.

The first major effect of clonidine treatment was to decrease choice variability, thus extending the causal role of noradrenaline to value-based decision-making. With increasing doses of clonidine, monkeys became increasingly more likely to repeat the same choice when presented with the same pairs of options throughout a session. This was confirmed by our model-based analysis, where we separated the evaluation from the option selection components (Padoa-Schioppa 2011). These two processes were controlled by three parameters: *k*_cost-benefit_ for the evaluation, *bias*, and *consistency* for the option selection. This model-based approach linked the behavioral change induced by clonidine with an increase of the consistency parameter, independent of the valuation process, and the bias parameter. Hence, clonidine induced an increase in choice consistency rather than a systematic bias of the choices in any direction (such as by reward, cost, or current vs. alternative option). This is in line with the results of three recent studies. First, specifically enhancing LC inputs to rat anterior cingulate cortex triggered behavioral variation (Tervo et al. 2014). Second, systemic clonidine injections increased decisiveness in rats performing a spatial decision-making task by reducing both the deliberative search process and the representation of the unchosen path in the hippocampus (Amemiya and Redish 2016). Finally, chemogenetic LC stimulation increased exploration in rats performing a patch-leaving task, though the effects were less specific than in our experiments, as LC stimulation also affected rats’ participation and performance (Kane et al. 2017). All together, this implies that the action of noradrenaline on choice consistency and potentially exploratory behavior is systematic and generic, rather than dependent upon specific task contingency. Thus, noradrenaline would promote adaptation mainly by making it more likely that animals randomly stumble into a novel option.

Variability in choices is assumed in many models of decision-making, but its functional role remains debated. It has been proposed that it arises from random noise driven by internal neural variability (Wang 2002; Faisal et al. 2008; Drugowitsch et al. 2016), and in that case, noradrenaline would control the amount of noise (Aston-Jones and Cohen 2005). Note that we chose to reason in terms of noradrenergic system activity, because clonidine has been shown to decrease LC firing rate, rather than shift its tonic/phasic firing mode. Moreover, we and others have failed to observe these modes, even in learning tasks (Bouret and Sara 2004; Bouret and Richmond 2015; Kalwani et al. 2014; Joshi et al. 2016). In a constant and deterministic environment, such as in our task, choices are optimal when they follow exactly the values of the options. But in uncertain and dynamic environments, it could be beneficial to try alternative options, should an unexpectedly better alternative appear. In that frame, noradrenaline is thought to control the adaptation to changes in action-outcome contingencies (Bouret and Sara 2005; Aston-Jones and Cohen 2005; Yu and Dayan 2005; Cohen et al. 2007; Nassar et al. 2012; Wilson et al. 2014). Hence, the clonidine-induced increase in choice consistency might decrease efficacy (reward rate) if animals were in a labile or uncertain environment, by hampering the ability to explore potentially better options.

The effect of clonidine on choice consistency was associated with an increase in reaction times, which we called a “speed-consistency trade-off.” In the frame of a drift diffusion model, this effect could be captured by the noise in the accumulation process or the setting of the decision boundaries, factors which are distinguishable by their effects on the variance of decision times. However, our task was not designed to capture decision times as we imposed a waiting period before the monkeys could respond, and our analyses showed that we could not reliably tease apart these two factors (data not shown). Nonetheless, this may be an elegant way in future studies to capture the effects of noradrenaline manipulations described here.

In line with the conclusions of our recent electrophysiological studies (Bouret and Richmond 2015; Varazzani et al. 2015), our direct manipulation of the noradrenergic system also highlighted its causal importance for effort processes. Clonidine dose-dependently reduced the amount of force produced, independent of the influence on reaction time, ruling out a global motor impairment. Moreover, it is also unlikely to be caused by a simple effect on arousal or vigilance, as clonidine had no impact on either the animals’ willingness to work or their ability to switch to the alternative option. The influence of clonidine on force production was also independent of task conditions. Indeed, the amount of force produced was not contingent in this task, and the effect of clonidine was equivalent across reward conditions. Hence, clonidine did not affect incentive processes, as it is often the case with dopaminergic treatments (Denk et al. 2005; Le Bouc et al. 2016; Yohn et al. 2016; Zénon et al. 2016).

Instead, the effect of clonidine on force production seems to be relatively specific to action production, but not necessarily to action initiation or persistence through the sequence as neither the initial choice to engage with the sequence nor the choice in the middle of the sequence was affected by the treatment. At first, this might appear surprising since sequences of actions are often considered to be an effort. But since the minimal force to validate a squeeze was very small and monkeys always succeeded if they initiated the action, physical exertion is unlikely to be a major component of the cost of each trial compared to the time over which the animal had to persist to gain the reward (Minamimoto et al. 2012). This is also in line with recent electrophysiological data in an effort-reward trade-off task showing an activation of LC neurons correlated with the amount of force produced on a grip at the time of executed action, but not when evaluating this option (Varazzani et al. 2015). This, together with work by Kalwani et al. (2014) showing a stronger relation between LC activation and action initiation rather than to decision-making, reinforces the idea that noradrenaline plays a specific role in actually producing the effort, mobilizing energy to face a challenge (Bouret and Richmond 2015; Varazzani et al. 2015). It is intriguing that such a role is complementary yet distinct from the influence of the other major catecholamine, dopamine, which is known to be a key for assessing the value of working through sequences of actions for reward but is perhaps not required to overcome force constraints (Ishiwari et al. 2004; Gan et al. 2010; Pasquereau and Turner 2013; Varazzani et al. 2015; Salamone et al. 2016). A key question for future studies will be to directly contrast the precise roles these neurotransmitters play in effort-based decision-making.

Last, we found that the effects on force production were not correlated with choice consistency, suggesting that noradrenaline plays specific, and at least partially separable, roles in behavioral flexibility and effort motivation. One possibility is that this could be mediated by two sets of LC target networks. Indeed, recent studies in rodents indicate that distinct LC neurons could target specific forebrain regions and support separate behavioral functions (Chandler et al. 2014; Uematsu et al. 2017). It may also relate to the fact that, as well as influencing alpha-2 auto-receptors and reducing central noradrenaline levels (Kawahara et al. 1999; Fernández-Pastor et al. 2005), clonidine also stimulates post-synaptic alpha-2 receptors and thus can alter synaptic transmission in, for example, prefrontal cortex. Note, however, the doses that we used are below those that affect working memory functions in monkeys, which was interpreted as clonidine-only post-synaptic alpha-2 receptors at higher doses (Franowicz and Arnsten 1999).

To conclude, these results demonstrate specific, complementary causal roles for noradrenaline in choice variability and motivation. Nonetheless, both functions might be subsumed under the overarching notion that noradrenaline plays a central role when facing challenges by (i) promoting an increase in behavioral volatility and (ii) mobilizing physical resources to respond to immediate challenges. This proposal relies on the assumption that these two processes are adaptive to solve most challenges. While the former would facilitate adaptation in uncertain or changing environments, the latter is clearly advantageous in environments where you must compete for resources and achieve the goals that are set.

## Electronic supplementary material

ESM_1 Subjects’ parameters’ estimate. Subjects’ k_cost-benefit_, bias and consistency parameter estimates for each treatment condition in the simple choice model. Color code corresponds to treatment condition. Error bars correspond to the uncertainty of parameters’ estimate under variational Bayes approach to model fitting. (PNG 31 kb)

High resolution image (TIFF 1519 kb)

ESM_2 Subjects’ choice curves. Probability to take the alternative option depending on the value of the current option and the corrected value of the alternative option (V(alternative option)* = V(alternative option) + bias). Color code corresponds to treatment condition. Top curves are *the choices computed from the model: p*robability to take the alternative option computed with the choice model (estimates of the consistency parameter). Bottom curves are the c*hoices computed from data:* the probability to take the alternative option is computed with subjects’ actual choices for the estimated values. Curves on the left correspond to monkeys A’s choice, curves in the middle to monkey D’s choices and curves on the right to monkey E’s choices. (PNG 53 kb)

High resolution image (TIFF 1519 kb)

ESM_3 Subjects’ full model parameters’ estimate. Subjects’ k_cost-benefit_, bias, side bias, k_squeezes done_ consistency and k_evaluation_ parameter estimates for each treatment condition in the full choice model. Color code corresponds to treatment condition. Error bars correspond to the uncertainty of parameters’ estimate under variational Bayes approach to model fitting. (PNG 38 kb)

High resolution image (TIFF 1519 kb)
