# Correction to: Dual contributions of noradrenaline to behavioural flexibility and motivation

**DOI:** 10.1007/s00213-018-5018-1

**Published:** 2018-09-11

**Authors:** Caroline I. Jahn, Sophie Gilardeau, Chiara Varazzani, Bastien Blain, Jerome Sallet, Mark E. Walton, Sebastien Bouret

**Affiliations:** 1 Motivation, Brain and Behavior Team, Institut du Cerveau et de la Moelle Epinière, CNRS UMR 7225 - INSERM U1127 – UPMC UMR S 1127, Hôpital Pitié-Salpétrière, 47, Boulevard de l’Hôpital, 75013 Paris, France; 2 Sorbonne Paris Cité Universités, Université Paris Descartes, Frontières du Vivant, 75005 Paris, France; 3 Department of Experimental Psychology, Oxford, OX1 3UD UK; 4 Wellcome Centre for Integrative Neuroimaging, University of Oxford, Oxford, UK; 5 Institute for Translational Neuroscience of Paris IHU-A-ICM, 75013 Paris, France; 6 Centre d’Economie de la Sorbonne, Université Paris 1, 75013 Paris, France

**Correction to: Psychopharmacology**

10.1007/s00213-018-4963-z

The article Dual contributions of noradrenaline to behavioural flexibility and motivation, written by Caroline I. Jahn, Sophie Gilardeau, Chiara Varazzani, Bastien Blain, Jerome Sallet, Mark E. Walton, Sebastien Bouret was originally published electronically on the publisher’s internet portal (currently SpringerLink) on 11 July 2018 without open access.

With the author(s)’ decision to opt for Open Choice the copyright of the article changed on 06 September 2018 to © The Author(s) 2018 and the article is forthwith distributed under the terms of the Creative Commons Attribution 4.0 International License (http://creativecommons.org/licenses/by/4.0/), which permits use, duplication, adaptation, distribution and reproduction in any medium or format, as long as you give appropriate credit to the original author(s) and the source, provide a link to the Creative Commons license and indicate if changes were made.

