# Supplementary figures and images for: Dual contributions of noradrenaline to behavioural flexibility and motivation
Source: Psychopharmacology (Berl). 2018 Jul 11;235(9):2687–702. doi: 10.1007/s00213-018-4963-z (PMC6182595; doi:10.1007/s00213-018-4963-z)

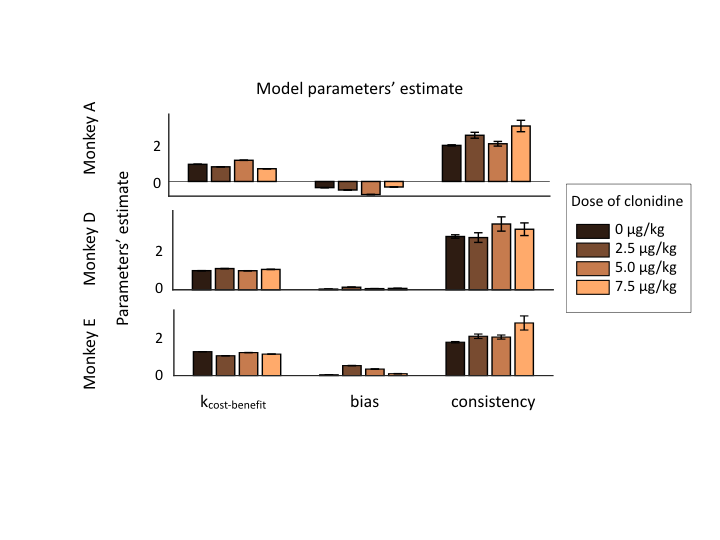

Supplement: Supplementary file 1 — Subjects’ parameters’ estimate. Subjects’ kcost-benefit, bias and consistency parameter estimates for each treatment condition in the simple choice model. Color code corresponds to treatment condition. Error bars correspond to the uncertainty of parameters’ estimate under variational Bayes approach to model fitting. (PNG 31 kb) [file 213_2018_4963_Fig7_ESM.png]

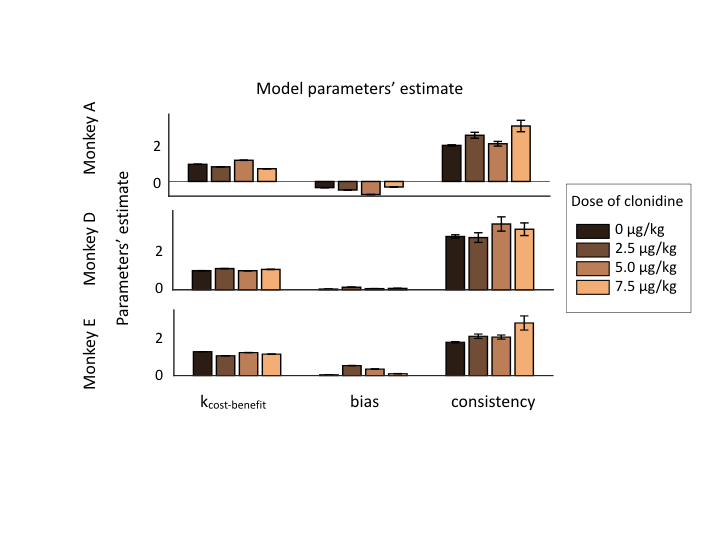

Supplement: Supplementary file 2 — High resolution image (TIFF 1519 kb) [file 213_2018_4963_MOESM1_ESM.tiff]

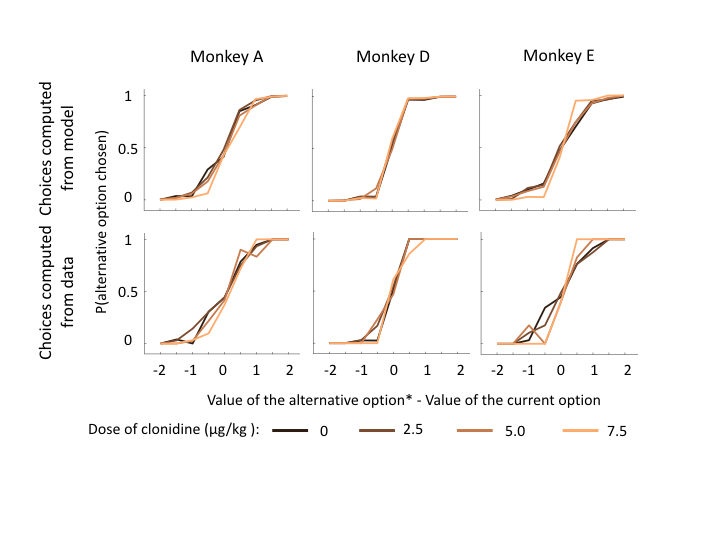

Supplement: Supplementary file 3 — Subjects’ choice curves. Probability to take the alternative option depending on the value of the current option and the corrected value of the alternative option (V(alternative option)* = V(alternative option) + bias). Color code corresponds to treatment condition. Top curves are the choices computed from the model: probability to take the alternative option computed with the choice model (estimates of the consistency parameter). Bottom curves are the choices computed from data: the probability to take the alternative option is computed with subjects’ actual choices for the estimated values. Curves on the left correspond to monkeys A’s choice, curves in the middle to monkey D’s choices and curves on the right to monkey E’s choices. (PNG 53 kb) [file 213_2018_4963_Fig8_ESM.png]

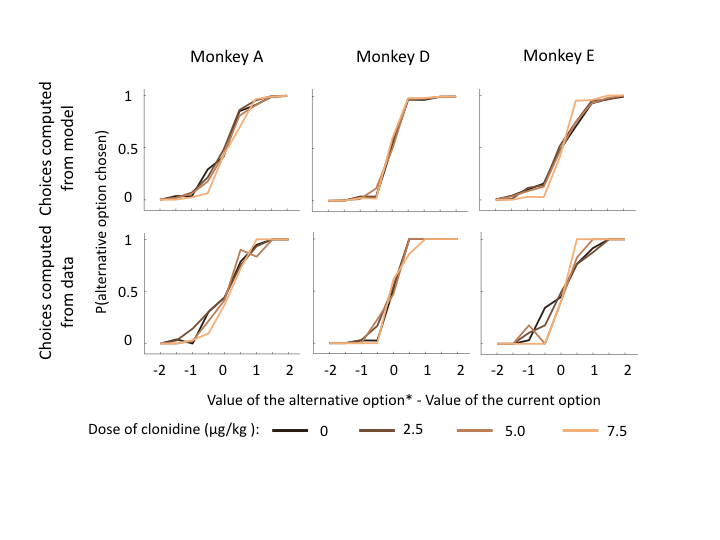

Supplement: Supplementary file 4 — High resolution image (TIFF 1519 kb) [file 213_2018_4963_MOESM2_ESM.tiff]

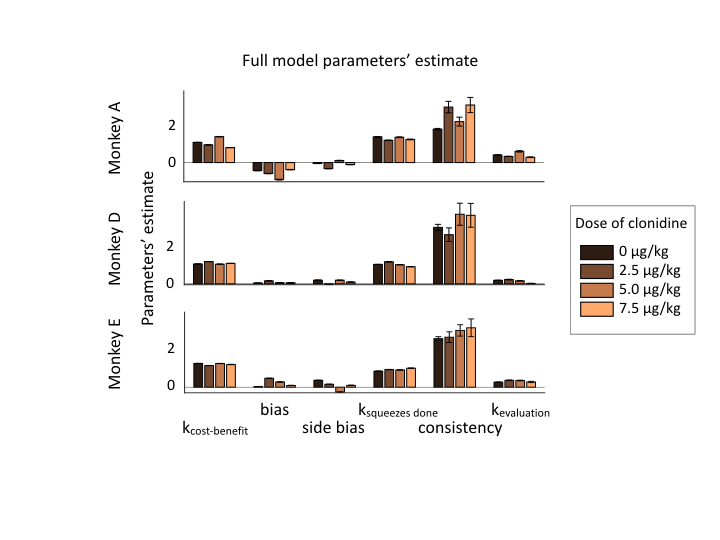

Supplement: Supplementary file 5 — Subjects’ full model parameters’ estimate. Subjects’ kcost-benefit, bias, side bias, ksqueezes done consistency and kevaluation parameter estimates for each treatment condition in the full choice model. Color code corresponds to treatment condition. Error bars correspond to the uncertainty of parameters’ estimate under variational Bayes approach to model fitting. (PNG 38 kb) [file 213_2018_4963_Fig9_ESM.png]

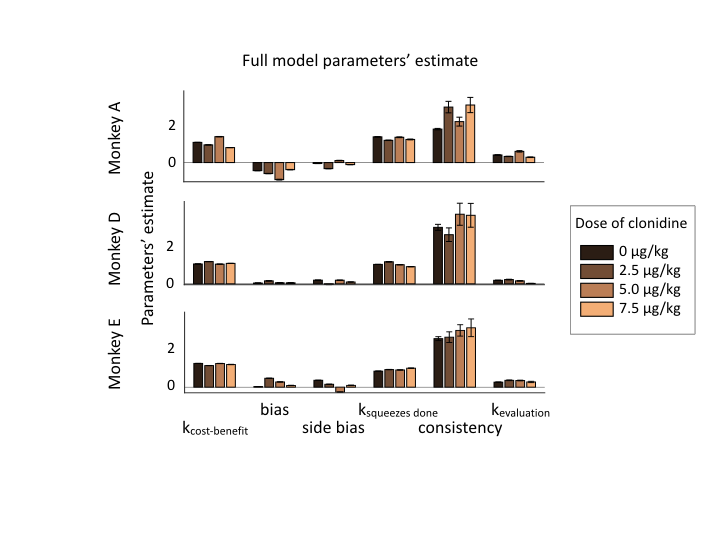

Supplement: Supplementary file 6 — High resolution image (TIFF 1519 kb) [file 213_2018_4963_MOESM3_ESM.tiff]
